# From Adiposity to Diastolic Dysfunction: The Integrated Role of Epicardial Fat, Autonomic Dysregulation, and Morning Blood Pressure Surge in Obesity

**DOI:** 10.1155/ijhy/8528281

**Published:** 2026-10-12

**Authors:** Ayhan Cosgun, Huseyin Oren

**Affiliations:** ^1^ Department of Cardiology TC Saglik Bakanligi Cihanbeyli Devlet Hastanesi Cihanbeyli Konya Türkiye; ^2^ Department of Cardiology TC Saglik Bakanligi Ankara Sehir Hastanesi Çankaya Ankara Türkiye

**Keywords:** diastolic dysfunction, epicardial adipose tissue, heart rate variability, morning blood pressure surge, obesity

## Abstract

**Objective:**

Although the association between obesity and left ventricular diastolic dysfunction (LVDD) is well established, the concurrent associations of epicardial adipose tissue (EAT), sleep‐trough (prewaking) morning blood pressure surge (MBPS), and autonomic imbalance with LVDD have not been fully elucidated. This study aimed to evaluate the associations of EAT thickness, MBPS, and heart rate variability (HRV) parameters with LVDD in obese adults and to assess the cross‐sectional discriminatory performance of these multimodal markers.

**Methods:**

This cross‐sectional study included 240 individuals, comprising 120 obese participants (body mass index ≥ 30 kg/m^2^) and 120 age‐ and sex‐matched normal‐weight controls (body mass index 18.5–24.9 kg/m^2^). All participants underwent transthoracic echocardiography, 24‐h ambulatory blood pressure monitoring, and 24‐h Holter electrocardiographic analysis, with HRV data available for the entire cohort (*n* = 240). EAT thickness was measured echocardiographically, and LVDD was classified using contemporary guideline‐based echocardiographic criteria. SDNN was predefined as the primary autonomic marker, while RMSSD and the LF/HF ratio were reported as supportive indices. Associations with LVDD (≥ Grade I) were evaluated using correlation analysis, multivariable logistic regression, and receiver operating characteristic analyses. Cross‐sectional discrimination analysis and performance assessment were performed within the obese subgroup (*n* = 120).

**Results:**

Compared with controls, obese participants exhibited greater EAT thickness (6.5 ± 1.7 vs. 4.0 ± 1.2 mm), higher MBPS (33 ± 10 vs. 21 ± 7 mmHg), increased LF/HF ratio, and lower SDNN and RMSSD values (all *p* < 0.001). The prevalence of LVDD was significantly higher in obese individuals than in controls (45% vs. 15%, *p* < 0.001). In multivariable analysis, EAT thickness (odds ratio [OR] = 1.20 per 1 mm; 95% confidence interval [CI] 1.08–1.34), MBPS (OR = 1.35 per 10 mmHg; 95% CI 1.10–1.65), and SDNN (OR = 0.88 per 10 ms; 95% CI 0.80–0.97) were independently associated with LVDD. The parsimonious model demonstrated discrimination and calibration comparable to or superior to the full clinical model (AUC: 0.89 vs. 0.85).

**Conclusion:**

In obese adults, increased epicardial adiposity, exaggerated MBPS, and autonomic dysfunction coexist with LVDD. Integrated assessment of structural (EAT), hemodynamic (MBPS), and autonomic (SDNN) markers may facilitate cross‐sectional identification of LVDD and improve phenotypic characterization in obesity.

**Highlights**
• In obese individuals, sleep‐trough (prewaking) morning blood pressure surge (MBPS) is significantly higher than in normal‐weight controls.
• Exaggerated MBPS is independently associated with left ventricular diastolic dysfunction (LVDD) in obese adults.
• Epicardial adipose tissue (EAT) thickness represents a structural marker associated with impaired diastolic function.
• Autonomic dysfunction is prominent in obesity; lower SDNN is inversely associated with the presence of LVDD.
• The combined evaluation of EAT, MBPS, and SDNN showed exploratory cross‐sectional discrimination for prevalent LVDD in individuals with obesity.
• The parsimonious model summarizes the concurrent associations of echocardiographic, ABPM‐derived, and Holter‐derived markers with prevalent LVDD.

## 1. Introduction

Obesity is a leading determinant of cardiovascular morbidity and mortality and, given its rising global prevalence, remains a major public health challenge in contemporary cardiology practice [1]. The increased frequency of hypertension, coronary artery disease, atrial fibrillation, and heart failure with preserved ejection fraction (EF) among obese individuals is attributable not only to excess hemodynamic load but also to interconnected pathophysiologic mechanisms including neurohormonal activation, subclinical systemic inflammation, autonomic nervous system imbalance, and ectopic fat deposition [2].

Within this spectrum, left ventricular diastolic dysfunction (LVDD) is regarded as an early phenotype of obesity‐related cardiac involvement [3]. Over time, diastolic dysfunction promotes left atrial remodeling and increases susceptibility to heart failure with preserved EF and atrial arrhythmias, even before overt systolic impairment becomes evident [4].

Epicardial adipose tissue (EAT) is a metabolically active visceral fat depot located in direct anatomic continuity with the myocardium and coronary arteries [1]. Through the secretion of adipokines, proinflammatory cytokines, and free fatty acids, EAT influences myocardial structure and function through paracrine and vasocrine signaling pathways [6]. Its close spatial proximity to intrinsic cardiac autonomic ganglia further suggests a role in the modulation of cardiac autonomic tone [7] and electrophysiologic stability [8].

Increased EAT thickness has been linked to coronary atherosclerosis, atrial fibrillation, ventricular arrhythmia risk, and adverse heart failure phenotypes, indicating that this tissue represents more than a passive fat store [9]. Instead, EAT appears to function as an active biologic compartment capable of shaping myocardial phenotype and cardiovascular risk [10].

In routine clinical practice, EAT thickness can be reliably measured using transthoracic echocardiography, allowing practical and reproducible risk assessment without radiation exposure or additional cost [11]. This feasibility makes EAT particularly attractive for integration into everyday cardiometabolic evaluation [12].

Although the association between obesity and diastolic dysfunction is well established, the combined association of epicardial adiposity, autonomic impairment, and circadian hemodynamic stress with prevalent diastolic dysfunction remains incompletely understood [13]. These domains are frequently studied in isolation, limiting insight into their potential convergence within a shared multimodal framework [14].

Heart rate variability (HRV) provides a noninvasive index of cardiac autonomic modulation, reflecting sympathovagal balance through time domain and frequency domain parameters [15]. Numerous studies have demonstrated reductions in SDNN and RMSSD in obese individuals, findings consistent with reduced parasympathetic activity [16]. In parallel, increases in the LF/HF ratio have been reported, reflecting a shift toward sympathetic predominance and autonomic imbalance [17].

Circadian blood pressure variability represents another dimension of cardiovascular risk that is not captured by conventional office or mean ambulatory measurements [18]. In particular, the morning blood pressure surge (MBPS) reflects the abrupt increase in systolic blood pressure (SBP) occurring during the transition from sleep to awakening [19]. Exaggerated MBPS has been independently associated with stroke, acute coronary events, and cardiovascular mortality [20].

In obesity, heightened sympathetic activity, renin–angiotensin–aldosterone system activation, and increased arterial stiffness may amplify the magnitude of the MBPS [21]. Repeated exposure to early morning hemodynamic loading may contribute to progressive myocardial stiffening and impaired ventricular relaxation [22].

The American Society of Echocardiography and European Association of Cardiovascular Imaging 2016 recommendations advocate a standardized multiparametric approach for classification of diastolic function incorporating mitral inflow indices, tissue Doppler e′ velocities, the E/e′ ratio, tricuspid regurgitation velocity, and left atrial volume index. This framework provides a robust reference standard for evaluating diastolic dysfunction in both clinical and research settings [23].

Accordingly, this study aimed to compare EAT thickness, sleep‐trough prewaking MBPS, 24‐h HRV indices, and left ventricular diastolic function between obese adults and age‐ and sex‐matched normal‐weight controls [24]. Within the obese subgroup, we additionally sought to evaluate the independent associations of EAT and MBPS with autonomic indices and diastolic dysfunction [25].

We hypothesized that increased EAT thickness and exaggerated MBPS are associated with a higher prevalence of diastolic dysfunction in obese individuals in conjunction with impaired autonomic modulation. We further hypothesized that these relationships persist independently of traditional markers such as body mass index and mean blood pressure [26].

To our knowledge, this is the first study to simultaneously integrate echocardiographic EAT thickness, ambulatory MBPS, and 24‐h HRV parameters within a unified analytical framework to identify *prevalent diastolic dysfunction* in obesity [9].

## 2. Materials and Methods

### 2.1. Study Design and Population

This study used a cross‐sectional, observational design. A total of 260 adults were screened for eligibility (screened cohort). Candidates meeting age (18–65 years) and BMI criteria were preassessed for inclusion in the obese (BMI ≥ 30 kg/m^2^) and normal‐weight control (BMI 18.5–24.9 kg/m^2^) groups. During screening, 20 individuals were excluded due to prespecified exclusion criteria or inadequate test quality (e.g., insufficient transthoracic echocardiographic image quality, technical inadequacy in Holter ECG or ambulatory blood pressure recordings, excessive artifact, or frequent ectopy). These exclusions occurred at the screening stage, yielding a final analytic cohort of 240 participants. The final cohort consisted of 120 obese participants and 120 normal‐weight controls individually matched for age (± 3 years) and sex. All 240 participants completed transthoracic echocardiography, 24‐h ambulatory blood pressure monitoring (ABPM), and 24‐h Holter ECG recordings per protocol with technically adequate quality.

#### 2.1.1. Inclusion Criteria

A total of 240 adults aged 18–65 years were enrolled at a tertiary cardiology center: 120 obese individuals (BMI ≥ 30 kg/m^2^) and 120 normal‐weight controls (BMI 18.5–24.9 kg/m^2^). Controls were matched to the obese group by age (± 3 years) and sex. Obese cases were recruited from obesity and cardiology outpatient clinics; controls were recruited from hospital staff and community volunteers.

#### 2.1.2. Exclusion Criteria

Known coronary artery disease, prior myocardial infarction or revascularization, heart failure, left ventricular EF < 50%, moderate‐to‐severe valvular disease, atrial fibrillation or frequent ventricular/supraventricular premature beats, secondary hypertension, insulin‐requiring diabetes mellitus (non–insulin‐requiring DM was included), chronic kidney disease (eGFR < 60 mL/min/1.73 m^2^), chronic inflammatory or systemic disease, antiarrhythmic therapy likely to substantially affect HRV, initiation of high‐dose beta‐blocker therapy within the past 3 months, and inadequate echocardiographic or Holter/ABPM quality were exclusionary.

All participants provided written informed consent. The study protocol was approved by the hospital ethics committee (Ethics Committee No: TABED‐15‐25) and the study was conducted in accordance with the Declaration of Helsinki.

### 2.2. Clinical and Laboratory Assessment

Demographics, cardiovascular risk factors, available medication information, smoking history, and sleep–wake times were recorded using a structured questionnaire. Anthropometric measures included height, weight, BMI, and waist circumference. Office blood pressure was measured using an automated device in the seated position after 10 min of rest; three measurements were taken 1–2 min apart and averaged. Fasting blood samples were obtained in the morning for glucose, lipid profile, creatinine, and routine biochemical parameters. *Hypertension and diabetes mellitus status were available and were included in the extended multivariable model. However, detailed information on antihypertensive medication classes and administration timing, HbA1c levels, and the use of SGLT2 inhibitors and GLP-1 receptor agonists was not systematically available and therefore could not be incorporated into the analyses.*

### 2.3. ABPM and MBPS

All participants underwent 24‐h ABPM using validated oscillometric devices in accordance with the European Society of Hypertension recommendations [24, 25]. Measurements were taken every 20–30 min during daytime hours and every 30–60 min at night. Sleep and wake periods were defined based on participant diaries. Recordings with ≥ 80% valid measurements and at least one measurement per hour were analyzed. Mean 24‐h blood pressure and daytime and nighttime SBP/DBP values were calculated. MBPS was defined as: MBPS = mean SBP during the first 2 h after awakening − mean SBP during the last 2 h before awakening.

Consistent with Kario’s work and subsequent studies, ≥ 35 mmHg was considered an “exaggerated morning surge” [18–22]. Dipping was not treated as a primary analytical variable in this study, given the mechanistic focus on MBPS as a dynamic marker of circadian blood pressure variability and sympathetic activation.

### 2.4. Holter Monitoring and HRV Analysis

All participants underwent 24‐h Holter ECG recording using three‐channel devices with a sampling rate of 1000 Hz. Recordings were analyzed by experienced technicians blinded to clinical data. After artifact and ectopy correction, normal‐to‐normal (NN) intervals were extracted and HRV was analyzed according to Task Force recommendations [15, 27]. Time‐domain indices included SDNN, SDANN, RMSSD, and pNN50; frequency‐domain indices included LF, HF, and the LF/HF ratio. HRV analysis followed predefined acceptability criteria, including sinus rhythm, adequate analyzable NN intervals, and at least 18 h of interpretable recording duration. Participants with technically inadequate Holter recordings were excluded at screening. Ultimately, HRV was available in the entire cohort (*n* = 240). For analytic modeling, SDNN was prioritized as the main representative of global autonomic function; RMSSD and LF/HF were reported in descriptive and supportive analyses.

### 2.5. Echocardiography and EAT Measurement

Transthoracic echocardiography was performed using a standard system equipped with a 2.5–3.5‐MHz transducer. All examinations were conducted and interpreted by cardiologists blinded to ABPM and HRV results. Two‐dimensional, M‐mode, Doppler, and tissue Doppler images were acquired according to ASE/EACVI recommendations [23]. LV EF was calculated using the biplane Simpson method; LV mass index and left atrial volume index (LAVI) were measured using standard methods. LV diastolic function was classified using the integrated 2016 ASE/EACVI algorithm (normal; Grades I–III LVDD) based on mitral inflow patterns, tissue Doppler e′ velocities, mean E/e′ ratio, TR velocity, and LAVI [23]. EAT thickness was measured in the parasternal long‐axis view as the echo‐lucent space between the outer wall of the right ventricle and the visceral pericardium at end‐systole; the mean of the maximum values across three consecutive beats was used for analysis [8–11]. End‐systolic measurement was selected to minimize underestimation due to diastolic compression and to align with commonly used echocardiographic protocols. To assess measurement reproducibility, blinded measurements were performed by two cardiologists in a randomly selected subsample of 30 cases and evaluated using the intraclass correlation coefficient (ICC = 0.92; 95% CI 0.89–0.96; *p* < 0.001).

### 2.6. Definitions and Endpoints

The primary endpoint was the presence of LVDD (grade ≥ I) according to ASE/EACVI criteria. The main independent variables were EAT thickness (continuous), MBPS (continuous and categorical at ≥ 35 mmHg), and HRV indices (especially SDNN). Secondary endpoints included the E/e′ ratio and LAVI.

### 2.7. Statistical Analysis

Statistical analyses were performed using SPSS v25.0 (IBM, Armonk, NY, USA). Continuous variables are presented as mean ± SD or median (IQR) depending on distribution; categorical variables are presented as counts and percentages. Normality was assessed using the Shapiro–Wilk test. Between‐group comparisons used the independent‐samples *t* test or Mann–Whitney *U* test for continuous variables and chi‐square or Fisher’s exact test for categorical variables. In the obese group, associations of EAT, MBPS, and obesity indices with HRV and diastolic parameters were assessed using Pearson’s or Spearman’s correlation as appropriate. Multivariable linear regression models were constructed for E/e′ and LAVI including age, sex, BMI, 24‐h mean SBP, EAT, and MBPS. Logistic regression analyses for LVDD (grade ≥ I) were performed at three levels: (i) minimally adjusted model (adjusted for age, sex, BMI, and 24‐h mean SBP; with EAT, MBPS, and SDNN as main variables of interest), (ii) extended clinical (full) model (minimally adjusted model plus DM, HT, and LDL), and (iii) parsimonious model (EAT, MBPS, SDNN). Model performance was evaluated using AUC, calibration slope, calibration intercept, and Brier score; internal validation and optimism correction were performed using 1000 bootstrap samples. Two‐sided *p* < 0.05 was considered statistically significant. In addition to regression‐based modeling, we evaluated a prespecified, rule‐based cutoff combination approach (EAT ≥ 5.5 mm, sleep‐trough MBPS ≥ 35 mmHg, SDNN < 120 ms) to enhance bedside interpretability. The methodological details and bootstrap‐based internal validation procedures for this rule‐based classifier are provided in Supporting Information S1.

The full model was constructed to comprehensively control for potential confounding by incorporating established clinical covariates alongside EAT, MBPS, and HRV parameters, thereby allowing robust assessment of independent associations with LVDD. In contrast, the reduced model was intentionally derived to enhance *interpretability* and parsimony by retaining only the strongest and most stable *markers*, minimizing overfitting and *supporting exploratory cross-sectional evaluation*. Bootstrap‐based optimism correction was applied to both models to ensure internally validated estimates of discrimination and calibration.

## 3. Results

### 3.1. Participants and Baseline Clinical Characteristics

A total of 240 individuals were included: 120 in the obese group and 120 age‐ and sex‐matched normal‐weight controls. There were no significant between‐group differences in age or sex distribution. In contrast, adiposity indices (BMI and waist circumference) and ambulatory blood pressure parameters were markedly higher in the obese group. Mean 24‐h systolic/diastolic blood pressure values were 130 ± 10/82 ± 8 mmHg in the obese group and 120 ± 8/75 ± 6 mmHg in controls (both *p* < 0.001). Sleep‐trough MBPS was also higher in obese participants (33 ± 10 mmHg vs. 21 ± 7 mmHg; *p* < 0.001) (Table 1).

**TABLE 1:** Demographic and baseline clinical characteristics of participants.

Variable	Control (*n* = 120) mean ± SD	Obese (*n* = 120) mean ± SD	Mean difference (obese − control)	95% CI	*t* (df = 238)	*p* value	Cohen’s *d*
Age (years)	48 ± 8	49 ± 8	+1.0	−1.0–3.0	0.84	0.40	0.13
BMI (kg/m^2^)	23.5 ± 1.8	33.0 ± 3.2	+9.5	8.83–10.17	27.9	< 0.001	3.65
Waist circumference (cm)	84 ± 7	108 ± 9	+24.0	21.96–26.04	23.1	< 0.001	2.99
Heart rate (bpm)	77.2 ± 6.2	84.2 ± 7.5	+7.0	5.25–8.75	7.87	< 0.001	1.02
24‐h SBP (mmHg)	120 ± 8	130 ± 10	+10.0	7.71–12.29	8.55	< 0.001	1.10
24‐h DBP (mmHg)	75 ± 6	82 ± 8	+7.0	5.21–8.79	7.69	< 0.001	0.99
MBPS (mmHg)	21 ± 7	33 ± 10	+12.0	9.83–14.17	10.8	< 0.001	1.39

*Note:* Continuous variables are presented as mean ± standard deviation (SD). Mean differences are expressed as obese minus control. Between‐group comparisons were performed using independent‐samples *t* tests. Effect sizes were quantified using Cohen’s *d*. MBPS, morning blood pressure surge (sleep‐trough definition).

Abbreviations: BMI, body mass index; BP, blood pressure; DBP, diastolic blood pressure; SBP, systolic blood pressure.

### 3.2. EAT, Autonomic Function (HRV), and Diastolic Function Findings

Echocardiography showed significantly greater EAT thickness in obese participants (6.5 ± 1.7 mm vs. 4.0 ± 1.2 mm; *p* < 0.001). HRV analysis demonstrated lower SDNN and RMSSD and a higher LF/HF ratio in the obese group (all *p*s < 0.001). Diastolic function parameters were worse in obese participants: lower E/A ratio, lower septal and lateral e′ velocities, and higher mean E/e′ ratio and LAVI (all *p* < 0.001). LVDD prevalence was 45.0% in obese participants and 15.0% in controls (*p* < 0.001) (Table 2).

**TABLE 2:** EAT, HRV, and diastolic function parameters (complete calculations).

Variable	Control (*n* = 120) mean ± SD	Obese (*n* = 120) mean ± SD	Mean difference	95% CI	*t*/*χ* ^2^	*p*	Cohen’s *d*
EAT thickness (mm)	4.0 ± 1.2	6.5 ± 1.7	+2.5	2.12–2.88	12.9	< 0.001	1.66
SDNN (ms)	150 ± 35	125 ± 30	−25	−33.2 to−16.8	−6.2	< 0.001	0.77
RMSSD (ms)	32 ± 10	24 ± 9	−8	−10.4 to−5.6	−6.6	< 0.001	0.84
LF/HF ratio	1.6 ± 0.6	2.3 ± 0.8	+0.7	0.53–0.87	7.7	< 0.001	0.99
Mitral E/A ratio	1.2 ± 0.3	0.9 ± 0.2	−0.3	−0.37 to−0.23	−9.3	< 0.001	1.17
Septal e′ (cm/s)	8.0 ± 1.5	6.5 ± 1.3	−1.5	−1.87 to−1.13	−8.2	< 0.001	1.06
Lateral e′ (cm/s)	10.5 ± 1.8	8.8 ± 1.7	−1.7	−2.16 to−1.24	−7.3	< 0.001	0.97
Mean e′ (cm/s)	9.3 ± 1.5	7.6 ± 1.4	−1.7	−2.08 to−1.32	−9.0	< 0.001	1.17
Mean E/e′	8.5 ± 1.8	11.5 ± 2.7	+3.0	2.42–3.58	9.2	< 0.001	1.33
LAVI (mL/m^2^)	26 ± 6	34 ± 8	+8.0	6.20–9.80	8.7	< 0.001	1.14
LVDD ≥ Grade I, *n* (%)	18 (15.0)	54 (45.0)	—	—	*χ* ^2^ = 25.7	< 0.001	OR≈4.6

*Note:* Continuous variables are presented as mean ± standard deviation. Mean differences are expressed as obese minus control. Between‐group comparisons were performed using independent‐samples *t* tests. Effect sizes are reported as Cohen’s *d*. Categorical variables were compared using the chi‐square test. HRV was available in the entire cohort (*n* = 240). SDNN, standard deviation of normal RR intervals; RMSSD, root mean square of successive RR differences; LF/HF, low‐frequency/high‐frequency ratio.

Abbreviations: EAT, epicardial adipose tissue; LAVI, left atrial volume index.

### 3.3. Correlation Analyses (Obese Group Only)

In the obese group (*n* = 120), higher global adiposity indices (BMI and waist circumference) were negatively correlated with SDNN and RMSSD and positively correlated with LF/HF, E/e′, and LAVI. EAT thickness showed similar significant correlations with markers of autonomic dysfunction and diastolic impairment. Sleep‐trough (prewaking) MBPS was inversely correlated with SDNN and RMSSD and positively correlated with LF/HF, E/e′, and LAVI (Table 3).

**TABLE 3:** Correlations in the obese group (*n* = 120) between EAT, morning BP surge, obesity indices, HRV, and diastolic function parameters.

Independent variable	SDNN, ms r (*p*)	RMSSD, ms r (*p*)	LF/HF r (*p*)	E/e′ r (*p*)	LAVI, mL/m^2^ r (*p*)
BMI, kg/m^2^	−0.30 (0.001)	−0.28 (0.002)	0.22 (0.015)	0.26 (0.004)	0.24 (0.007)
Waist circumference, cm	−0.32 (< 0.001)	−0.27 (0.003)	0.24 (0.009)	0.28 (0.002)	0.26 (0.004)
EAT thickness, mm	−0.27 (0.003)	−0.25 (0.006)	0.29 (0.002)	0.40 (< 0.001)	0.35 (< 0.001)
Sleep‐trough (prewaking) MBPS, mmHg	−0.20 (0.030)	−0.18 (0.045)	0.25 (0.006)	0.35 (< 0.001)	0.25 (0.006)

*Note:* Correlation analyses were performed within the obese subgroup (*n* = 120). Pearson’s or Spearman’s correlation coefficients were applied as appropriate based on variable distribution. SDNN, standard deviation of normal RR intervals. RMSSD, root mean square of successive RR interval differences; LF/HF, low‐frequency to high‐frequency ratio; MBPS, morning blood pressure surge (sleep‐trough definition).

Abbreviations: BMI, body mass index; EAT, epicardial adipose tissue; LAVI, left atrial volume index.

### 3.4. Multivariable Logistic Regression (LVDD ≥ Grade I in the Obese Group)

In the obese group (*n* = 120), with LVDD of at least Grade I as the dependent variable, EAT thickness, sleep‐trough (prewaking) MBPS, and SDNN remained *independently associated with LVDD* after adjustment for age, sex, BMI, and 24‐h SBP. Each 1 mm increase in EAT thickness was associated with higher odds of LVDD (OR = 1.20; 95% CI 1.08–1.34; *p* < 0.001), and each 10 mmHg increase in MBPS was associated with *higher odds of prevalent LVDD* (OR = 1.35; 95% CI 1.10–1.65; *p* = 0.003). Each 10 ms increase in SDNN was associated with lower odds of LVDD (OR = 0.88; 95% CI 0.80–0.97; *p* = 0.010) (Table 4).

**TABLE 4:** Multivariable logistic regression analysis for diastolic dysfunction (≥ Grade I) (obese group, *n* = 120): prespecified minimally adjusted model.

Variable	OR	95% CI	*p* value
Age, years (per 1‐year increase)	1.02	(0.99–1.05)	0.15
Female sex (male = reference)	0.90	(0.50–1.60)	0.72
BMI, kg/m^2^ (per 1 kg/m^2^ increase)	1.05	(0.98–1.13)	0.18
24‐h systolic BP, mmHg (per 1 mmHg increase)	1.02	(0.99–1.05)	0.16
EAT thickness, mm (per 1 mm increase)	1.20	(1.08–1.34)	< 0.001
Sleep‐trough (prewaking) MBPS, per 10 mmHg (per 10 mmHg increase)	1.35	(1.10–1.65)	0.003
SDNN, per 10 ms (per 10 ms increase)	0.88	(0.80–0.97)	0.010

*Note:* Multivariable logistic regression analysis examining factors associated with left ventricular diastolic dysfunction (LVDD ≥ grade I) in obese participants (*n* = 120). The minimally adjusted model included age, sex, body mass index (BMI), and 24‐h systolic blood pressure as potential confounders. Epicardial adipose tissue (EAT) thickness, morning blood pressure surge (MBPS), and SDNN were prespecified variables of interest. MBPS and SDNN are presented per 10‐unit increase. SDNN, standard deviation of normal RR intervals. Dependent variable: Diastolic dysfunction ≥ Grade I (absent = 0, present = 1).

Abbreviations: BP, blood pressure; CI, confidence interval; EAT, epicardial adipose tissue; MBPS, morning blood pressure surge; OR, odds ratio.

Table 4 presents the prespecified minimally adjusted model, whereas Table 5 presents the extended (full) model results.

**TABLE 5:** Factors associated with diastolic dysfunction (≥ Grade I) in the obese group (*n* = 120): univariable and multivariable logistic regression (full model).

Variable	Univariable OR (95% CI)	*p*	Multivariable OR (95% CI)	*p*
Age, years (per + 1 year)	1.04 (0.97–1.07)	0.117	1.01 (0.98–1.04)	0.51
Diabetes mellitus	1.82 (1.13–2.94)	0.014	1.66 (1.09–2.56)	0.02
Hypertension	1.52 (1.02–2.32)	0.045	1.48 (1.05–2.23)	0.048
LDL‐cholesterol, per 10 mg/dL	1.03 (0.88–1.22)	0.72	1.02 (0.91–1.20)	0.78
Female sex (male = ref)	0.93 (0.55–1.67)	0.79	0.89 (0.48–1.57)	0.70
BMI, kg/m^2^ (per + 1 kg/m^2^)	1.08 (0.98–1.15)	0.059	1.06 (0.98–1.17)	0.20
24‐h systolic BP, mmHg (per 1 mmHg)	1.02 (0.96–1.05)	0.61	1.01 (0.96–1.08)	0.74
EAT thickness, mm (per + 1 mm)	1.25 (1.06–1.41)	0.002	1.22 (1.05–1.33)	0.001
Sleep‐trough (prewaking) MBPS (per + 10 mmHg)	1.37 (1.14–1.77)	0.005	1.33 (1.08–1.63)	0.007
SDNN (per + 10 ms)	0.88 (0.79–0.98)	0.020	0.86 (0.78–0.96)	0.005

*Note:* Univariable and multivariable logistic regression analyses assessing clinical, metabolic, hemodynamic, and autonomic predictors of left ventricular diastolic dysfunction (LVDD ≥ grade I) in obese participants. Odds ratios (ORs) for continuous variables are reported per specified unit increment. Dependent variable: Diastolic dysfunction ≥ Grade I (absent = 0, present = 1). Units: For continuous variables, ORs are reported per specified unit increase. SDNN, standard deviation of normal RR intervals.

Abbreviations: BMI, body mass index; BP, blood pressure; CI, confidence interval; EAT, epicardial adipose tissue; LDL, low‐density lipoprotein cholesterol; MBPS, morning blood pressure surge; OR, odds ratio.

In our study, the loss of statistical significance of traditional clinical risk factors associated with LVDD (BMI, heart rate, LDL‐cholesterol) in multivariable models suggests that in obesity, diastolic dysfunction may be more strongly associated with proximal phenotypic markers. EAT thickness, MBPS, and SDNN—reflecting local adiposity, hemodynamic stress, and autonomic dysfunction—capture complementary domains and collectively enhance discrimination.

Although diabetes mellitus and hypertension remained statistically significant in the full multivariable model, the parsimonious reduced model was intentionally constructed to prioritize EAT thickness, sleep‐trough (prewaking) MBPS, and SDNN as core markers of interest (Table 5). The rationale for this variable selection was not to exclude established clinical risk factors, but to enhance *interpretability* and model parsimony by focusing on a limited set of readily measurable markers representing adiposity‐related, hemodynamic, and autonomic domains. This approach allowed optimization of model performance while preserving interpretability, without implying a diminished clinical relevance of diabetes mellitus or hypertension.

### 3.5. ROC Analysis (Cutoffs and Discriminatory Performance)

ROC analyses were performed in the obese subgroup *to assess cross-sectional discrimination*. In ROC analysis, EAT thickness showed the highest single‐marker performance for discriminating LVDD (≥ Grade I) (AUC = 0.82; 95% CI 0.71–0.87; *p* < 0.001); for EAT ≥ 5.5 mm, sensitivity was 77% and specificity 72%. For MBPS ≥ 35 mmHg, AUC was 0.78 (95% CI 0.70–0.85; *p* < 0.001) with sensitivity 71% and specificity 70%. For SDNN < 120 ms, AUC was 0.79 (95% CI 0.70–0.88; *p* < 0.001) with sensitivity 70% and specificity 71% (Table 6). Key performance metrics for the rule‐based approach are summarized in Table 6, with detailed internal validation results provided in the Supplement. Because the rule‐based approach produces a dichotomous classifier, its AUC (c‐statistic) corresponds to a single ROC operating point and is mathematically equivalent to balanced accuracy (sensitivity + specificity)/2. A detailed description of the rule definition, validation approach, and ROC representation for the dichotomous classifier is provided in Supporting Information S1 and Figure S1.

**TABLE 6:** ROC analysis in the obese subgroup (*n* = 120): discriminatory performance of clinical, hemodynamic, and autonomic parameters for LVDD.

Parameter	AUC (c‐statistic)	95% CI	Prespecified clinical threshold	Sensitivity (%)	Specificity (%)	Balanced accuracy^∗^	*p* value
Age (continuous)	0.62	0.58–0.72	> 60 years	56	59	0.58	0.016
24‐h SBP (mmHg)	0.66	0.61–0.73	> 130 mmHg	66	65	0.66	< 0.001
Epicardial adipose tissue (EAT, mm)	0.82	0.71–0.87	≥ 5.5 mm	77	72	0.75	< 0.001
Morning blood pressure surge (MBPS, mmHg)	0.78	0.70–0.85	≥ 35 mmHg	71	70	0.71	< 0.001
SDNN (ms)	0.79	0.70–0.88	< 120 ms	70	71	0.71	< 0.001
EAT + MBPS + SDNN (rule‐based cutoff model)	0.77	0.71–0.86	5.5/35/120	79	75	0.77	—

*Note:* Receiver operating characteristic (ROC) analyses were performed in the obese subgroup (*n* = 120). For single markers, AUCs were calculated using continuous variable forms, while sensitivity and specificity correspond to prespecified clinically meaningful thresholds. The rule‐based cutoff model represents a binary (0/1) classifier combining epicardial adipose tissue (EAT ≥ 5.5 mm), morning blood pressure surge (MBPS ≥ 35 mmHg), and SDNN (< 120 ms). For this dichotomous predictor, discrimination is expressed as the c‐statistic of a binary classifier, which under standard ROC linear interpolation is mathematically equivalent to balanced accuracy ([sensitivity + specificity]/2). As the rule‐based model does not generate a continuously varying score, its ROC representation corresponds to a single operating point and no *p* value is reported. AUC, area under the receiver operating characteristic curve. SDNN, standard deviation of normal RR intervals.

Abbreviations: EAT, epicardial adipose tissue; LVDD, left ventricular diastolic dysfunction; MBPS, morning blood pressure surge; SBP, systolic blood pressure.

^∗^Balanced accuracy = (sensitivity + spesifity)/2, with sensitivity and specifity expressed as proportions.

### 3.6. Model Performance (Full vs. Parsimonious) and Internal Validation

Model performance analyses were conducted in the obese subgroup (*n* = 120). At the model level, the parsimonious continuous model (EAT + MBPS + SDNN) was compared with the extended clinical model (clinical covariates + EAT + MBPS + SDNN). The full model had an AUC of 0.85, whereas the parsimonious continuous model had an AUC of 0.89; optimism‐corrected AUC values were 0.84 and 0.88, respectively. The calibration slope was higher in the parsimonious model (0.94 vs. 0.82), and the Brier score was lower (0.11 vs. 0.15). DeLong’s test compared the AUCs of the full versus reduced models (0.85 vs. 0.89). For correlated ROC curves, DeLong’s test showed that the AUC difference was statistically significant (ΔAUC = 0.04; *p* = 0.04). After bootstrap internal validation, the optimism‐corrected AUC of the parsimonious model was 0.88. The reduced model supports parsimony, but external validation is required to confirm the generalizability of these differences (Figure 1) (Table 6).

**FIGURE 1:**
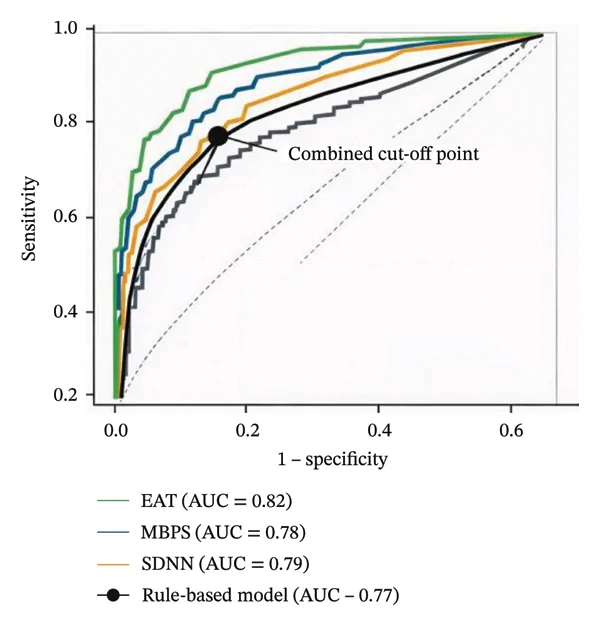
ROC analysis figure for LV diastolic dysfunction. *Note:* AUC, area under the receiver operating characteristic curve; SDNN, standard deviation of normal RR intervals. Abbreviations: SBP, systolic blood pressure; EAT, epicardial adipose tissue; MBPS, morning blood pressure surge; LVDD, left ventricular diastolic dysfunction.

#### 3.6.1. Model Performance

• AUC: 0.85 (full) vs. 0.89 (reduced)
• Optimism‐corrected AUC: 0.84 vs. 0.88
• Calibration slope: 0.82 vs. 0.94
• Brier score: 0.15 vs. 0.11
• Intercept:0.07 vs. 0.05

#### 3.6.2. Internal Validation (Bootstrap) and Optimism‐Corrected Performance

In bootstrap internal validation, the reduced continuous model (logistic model using EAT + MBPS + SDNN as continuous *variables*) demonstrated *cross-sectional discriminatory performance* with an optimism‐corrected AUC of 0.88, compared with 0.84 for the full model. The reduced model also showed moderately better performance, with a higher calibration slope (0.94 vs. 0.82) and lower *classification error* as reflected by the Brier score (0.11 vs. 0.15). Calibration‐in‐the‐large (intercept) was close to zero, indicating no evidence of systematic *probability* over‐ or underestimation. Unlike several previous studies that primarily reported discrimination metrics, we additionally reported calibration‐in‐the‐large to provide a more comprehensive assessment of model calibration (Calibration‐in‐the‐large [intercept]: 0.07 [full] vs. 0.05 [reduced]). Calibration intercept and slope were obtained from a logistic recalibration model with LVDD as the outcome and the model’s linear predictor as the covariate. For the complementary rule‐based classifier (binary cutoff combination), the bootstrap‐based optimism correction and percentile confidence intervals are reported in Supporting Results S1.

## 4. Discussion

### 4.1. Key Findings and Alignment With Prior Literature

In this cross‐sectional obese–control comparative study, we evaluated structural, hemodynamic, and autonomic domains within a single analytical framework and demonstrated that obese adults exhibit a higher prevalence of LVDD together with increased EAT thickness, more pronounced sleep‐trough MBPS, and impaired autonomic modulation as reflected by reduced HRV [4]. Within the obese subgroup, multivariable logistic regression analysis further showed that EAT thickness, MBPS, and SDNN retained independent associations with diastolic dysfunction, supporting the concept that obesity‐related diastolic impairment reflects concurrent abnormalities across multiple domains rather than hemodynamic load alone [3].

The substantially higher prevalence of diastolic dysfunction observed among obese individuals compared with normal‐weight controls is consistent with previous reports demonstrating that obesity is associated with early impairment in myocardial relaxation and filling properties even in the absence of overt heart failure [3]. These findings reinforce the view that diastolic dysfunction represents a subclinical but clinically meaningful manifestation of obesity‐related myocardial involvement that may precede the development of heart failure with preserved EF [4]. Importantly, the observed prevalence aligns with prior community‐based and clinical cohort studies, supporting the external plausibility of our findings [28].

### 4.2. EAT and Diastolic Impairment

EAT thickness was significantly greater in obese participants, and this increase was independently associated with indices of diastolic dysfunction [1]. EAT is increasingly recognized as an active visceral fat compartment that influences myocardial structure and function through paracrine and vasocrine pathways [6]. Local secretion of proinflammatory cytokines, adipokines, and free fatty acids may promote myocardial fibrosis, oxidative stress, and impaired ventricular compliance, thereby contributing to elevated filling pressures and abnormal relaxation [2].

Several prior studies have demonstrated associations between EAT thickness and diastolic function parameters such as the E/e′ ratio and left atrial volume index in both hypertensive and community‐based populations [12]. Our findings extend this evidence by showing that EAT remains independently associated with diastolic dysfunction even after adjustment for traditional cardiometabolic risk factors within an obese cohort [10]. This suggests that epicardial adiposity may represent a marker associated with diastolic impairment rather than merely a surrogate marker of generalized obesity [9].

Recent evidence has further contextualized EAT within a broader cardiometabolic and biomarker framework. In patients with newly diagnosed type 2 diabetes, computed tomography–quantified EAT volume was independently associated with circulating GDF‐15 and soluble ST2 levels, while 12 months of metabolic optimization was accompanied by reductions in indexed EAT volume and soluble ST2, although GDF‐15 and galectin‐3 remained unchanged [29]. A recent translational review similarly highlighted the complementary roles of EAT imaging and circulating markers of myocardial stress, fibrosis, and inflammation in characterizing heart failure–related phenotypes in type 2 diabetes [30]. These observations provide additional biological context for EAT assessment but do not establish that circulating biomarkers originate specifically from EAT or mediate myocardial dysfunction.

### 4.3. Autonomic Dysfunction and Heart Rate Variability

Impaired autonomic modulation, reflected by reduced SDNN and RMSSD, was a prominent feature among obese participants in our study [13]. HRV provides a well‐established noninvasive marker of cardiac autonomic balance, with reductions in time‐domain indices indicating diminished parasympathetic tone and increased sympathetic predominance [15]. Numerous prior investigations have reported similar autonomic alterations in obesity, metabolic syndrome, and insulin resistance, linking autonomic imbalance to increased cardiovascular risk [16].

In our multivariable analysis, SDNN remained independently and inversely associated with diastolic dysfunction, suggesting that global autonomic impairment may be associated with abnormalities in ventricular relaxation [17]. Chronic sympathetic activation may increase heart rate, vascular tone, and neurohormonal stimulation, thereby imposing sustained hemodynamic and metabolic stress on the myocardium [14]. These findings support the concept that autonomic dysfunction represents a concurrent feature of the obesity‐related diastolic phenotype rather than a secondary epiphenomenon [27].

### 4.4. MBPS and Diastolic Loading

Sleep‐trough MBPS was significantly higher in obese individuals and independently associated with diastolic dysfunction [18]. MBPS reflects the abrupt increase in blood pressure during the transition from sleep to awakening and has been linked to target‐organ damage and adverse cardiovascular outcomes independent of mean blood pressure levels [19]. In obesity, heightened sympathetic activation, renin–angiotensin–aldosterone system upregulation, and increased arterial stiffness may amplify this surge [21].

Repeated exposure to exaggerated early‐morning hemodynamic loading may contribute to progressive myocardial stiffening and impaired ventricular relaxation over time [22]. Our findings extend prior observations by demonstrating that MBPS is associated with diastolic dysfunction in obese individuals even after adjustment for average ambulatory blood pressure, supporting an independent statistical association between circadian hemodynamic stress and obesity‐related cardiac dysfunction [20].

### 4.5. The EAT–MBPS–SDNN Framework as a Hypothesis‐Generating Multimodal Association

A central contribution of the present study is the integrated evaluation of EAT thickness, MBPS, and autonomic modulation within a unified analytical framework [8]. The persistence of independent associations for all three parameters suggests that they constitute a hypothesis‐generating multimodal framework associated with diastolic dysfunction in obesity [9]. This framework integrates visceral cardiac adiposity, circadian hemodynamic stress, and autonomic imbalance into a coherent conceptual model.

Importantly, this framework does not negate the relevance of conventional cardiometabolic comorbidities such as hypertension and diabetes mellitus [25]. Instead, it suggests that part of their deleterious impact on diastolic function may be statistically associated with these proximal markers [26]. From this perspective, EAT, MBPS, and autonomic dysfunction represent potentially relevant associated markers rather than purely descriptive *correlates* [4].

### 4.6. Rationale for Modeling Strategy and Parsimony

In constructing *cross-sectional models* within a cohort of limited size, careful balance between comprehensive adjustment and model parsimony is essential to minimize overfitting [23]. Accordingly, we evaluated both a full multivariable model incorporating demographic and cardiometabolic covariates and a reduced model focusing on the EAT–MBPS–SDNN triad. The reduced model demonstrated discrimination and calibration comparable to or exceeding those that of the full model, supporting its interpretability and potential generalizability [23].

These findings suggest that a compact set of multimodal markers may efficiently identify *prevalent diastolic dysfunction* in obese individuals [23]. While broader clinical models remain valuable, the reduced approach highlights the dominant statistical signals conveyed by epicardial adiposity, circadian hemodynamic stress, and autonomic imbalance [8].

### 4.7. Clinical Implications

From a clinical standpoint, combined assessment of EAT thickness, ambulatory MBPS, and Holter‐derived SDNN offers a practical and noninvasive strategy for *identifying obese individuals with prevalent subclinical diastolic dysfunction* [4]. This approach moves beyond static anthropometric or blood pressure measures and incorporates dynamic parameters from multiple physiological domains [18].

Weight reduction strategies may decrease EAT burden, lifestyle modification and physical conditioning may improve autonomic balance, and chronotherapeutic approaches may attenuate exaggerated MBPS [21]. Accordingly, the EAT–MBPS–autonomic framework represents a hypothesis‐generating approach for both phenotypic characterization and prospective investigation [4].

### 4.8. Limitations and Future Directions

Several limitations should be acknowledged. The cross‐sectional design and the absence of mediation or pathway analyses preclude causal inference or temporal prediction regarding the temporal relationships among EAT, autonomic dysfunction, MBPS, and diastolic impairment [24]. The single‐center nature of the study may limit generalizability, and EAT was assessed by echocardiography rather than volumetric imaging modalities [11]. Residual confounding from factors such as sleep apnea, medication timing, physical activity, and dietary habits cannot be fully excluded [22].

Future longitudinal and interventional studies are warranted to clarify whether modification of EAT burden, autonomic balance, or circadian blood pressure patterns can alter the trajectory of diastolic dysfunction and prevent progression toward heart failure with preserved EF [9].

Previous studies have reported reductions in EAT thickness or volume with SGLT2 inhibitors and GLP‐1 receptor agonists [31–35], while human EAT has been shown to express GLP‐1 and GLP‐2 receptor genes [36].

The absence of detailed information on antihypertensive medication classes and administration timing, HbA1c levels, and the use of SGLT2 inhibitors and GLP‐1 receptor agonists may have resulted in residual confounding, as these treatments may influence morning blood pressure patterns, autonomic parameters, cardiac function, and EAT burden.

## 5. Conclusion

In this study, LVDD in obese adults was closely associated with increased EAT thickness, exaggerated MBPS, and impaired autonomic modulation as reflected by reduced HRV. The persistence of independent associations for these three parameters indicates that obesity‐related diastolic impairment is concurrently associated with adiposity‐related, hemodynamic, and autonomic abnormalities rather than with excess body mass or mean blood pressure alone. Although the cross‐sectional design precludes causal inference, the consistent signals observed across structural, circadian, and autonomic domains support the clinical relevance of this integrated phenotype. From a practical perspective, the combined assessment of echocardiographic EAT, ambulatory MBPS, and Holter‐derived HRV provides a feasible and multimodal approach for *cross-sectional identification of prevalent diastolic dysfunction phenotypes* in obesity, warranting confirmation in prospective and interventional studies [9].

## Author Contributions

Study conception and design: Ayhan Cosgun.

Data collection: Ayhan Cosgun, Huseyin Oren.

Echocardiographic and ECG analysis: Ayhan Cosgun, Huseyin Oren.

Statistical analysis and interpretation: Ayhan Cosgun.

Manuscript drafting and revision: Ayhan Cosgun.

## Funding

This research received no specific grant from any funding agency in the public, commercial, or not‐for‐profit sectors.

## Disclosure

All authors reviewed and approved the final version of the manuscript.

## Conflicts of Interest

The authors declare no conflicts of interest.

## Supporting Information

Additional supporting information can be found online in the Supporting Information section.

## Supporting information


**Supporting Information** The Supporting Appendix includes Supporting Information S1, detailing the internal validation of the prespecified rule‐based (cutoff combination) model using bootstrap resampling, Supporting Results S1, reporting the *cross-sectional discriminatory performance* and optimism‐corrected discrimination of the rule‐based classifier in the obese subgroup, and Figure S1, illustrating the ROC representation of the dichotomous rule‐based model.

## Data Availability

The data supporting the findings of this study are available from the corresponding author upon reasonable request.
