# Supplementary material for: From Adiposity to Diastolic Dysfunction: The Integrated Role of Epicardial Fat, Autonomic Dysregulation, and Morning Blood Pressure Surge in Obesity
Source: Int J Hypertens. 2026 Oct 12;2026:8528281. doi: 10.1155/ijhy/8528281 (PMC13659054; doi:10.1155/ijhy/8528281)
Supplement: Supplementary file 1 [file IJHY-2026-8528281-s001.docx]

**Supplementary Methods S1:**

**Internal Validation of the Rule-Based (Cut-off Combination) Model**

In addition to regression-based analyses, we evaluated a complementary rule-based classification approach to enhance clinical interpretability by integrating prespecified, physiologically grounded thresholds spanning structural, hemodynamic, and autonomic domains.

**Rule definition (a priori)**

The rule-based model was defined a priori, without any data-driven optimization, using thresholds derived from prior literature and guideline-supported clinical practice:

- Epicardial adipose tissue (EAT) thickness **≥** 5.5 mm
- Sleep-trough morning blood pressure surge (MBPS) **≥** 35 mmHg
- Standard deviation of NN intervals (SDNN) **<** 120 ms

Participants meeting all three criteria were classified as rule-positive for the presence of LV diastolic dysfunction (LVDD ≥ grade I). The rule was intentionally constructed as a binary decision rule; no regression coefficients were estimated and no individual predicted probabilities were generated.

**Analysis population**

Because the rule requires technically adequate HRV assessment, the rule-based model was evaluated among participants with valid HRV recordings. Although HRV data were available in the full cohort (n = 240), diagnostic performance analyses for the rule were restricted to the obese subgroup (n = 120) to remain consistent with the study’s primary prediction-modeling population. HRV was available for all obese participants (n=120), therefore no additional exclusions were required for the rule-based analysis.

**Internal validation and optimism correction**

To assess internal validity and mitigate optimism related to sample-specific effects, we performed bootstrap resampling (1,000 iterations). In each bootstrap sample, the predefined rule was reapplied unchanged, yielding a binary score (0/1). Discrimination was quantified using the c-statistic (equivalent to the AUC) for a dichotomous predictor.

Optimism was estimated as the mean difference between performance in the bootstrap samples (apparent performance in each resample) and performance when the same rule was evaluated in the original dataset. The optimism-corrected AUC was obtained by subtracting mean optimism from the apparent (naïve) AUC.

**Supplementary Results S1. Performance of the Rule-Based Cut-off Model**

Within the obese subgroup (n = 120), the predefined rule-based cut-off model (EAT ≥ 5.5 mm, sleep-trough MBPS ≥ 35 mmHg, and SDNN < 120 ms) demonstrated clinically plausible discrimination for identifying LVDD (≥ grade I). The rule achieved **79% sensitivity and 75% specificity**, corresponding to a c-statistic (AUC) of **0.77**, which—because the rule is a **binary classifier**—is equivalent to **balanced accuracy** (0.79+0.75)/2. After bootstrap internal validation (1,000 resamples), the optimism-corrected c-statistic remained **0.77 (95% CI: 0.71–0.86).** 95% CI was obtained using bootstrap percentile CI .Optimism correction was derived from bootstrap resampling.Compared with single markers, the rule’s discrimination was numerically lower than EAT alone (AUC 0.82) but comparable to MBPS (AUC 0.78) and SDNN (AUC 0.79). As the rule operates at prespecified thresholds, its clinical utility is best reflected by its operating characteristics rather than marginal differences in global AUC.

**Figure S1. ROC Curve for the Rule-based Cut-off Combination Model**

**Figure S1. ROC Representation for the Rule-Based Cut-off Combination Model**

Receiver operating characteristic (ROC) representation of the predefined rule combining EAT thickness ≥ 5.5 mm, sleep-trough MBPS ≥ 35 mmHg, and SDNN < 120 ms for identifying LVDD (≥ grade I) in the obese subgroup. The optimism-corrected c-statistic (AUC) obtained by bootstrap internal validation was 0.77.

**Footnote:**
Because the rule yields a **binary classifier (0/1),** the ROC representation corresponds to a **single operating point**. The reported AUC is the **c-statistic for a dichotomous predictor**, i.e., the concordance probability, and is therefore appropriate for quantifying discrimination in this context.
